# A forgotten group during humanitarian crises: a systematic review of sexual and reproductive health interventions for young people including adolescents in humanitarian settings

**DOI:** 10.1186/s13031-019-0240-y

**Published:** 2019-11-27

**Authors:** Lauren Jennings, Asha S. George, Tanya Jacobs, Karl Blanchet, Neha S. Singh

**Affiliations:** 1 Health in Humanitarian Crises Centre, London School of Hygiene and Tropical Medicine, 15-17 Tavistock Place, London, WC1H 9SH UK; 2 School of Public Health, University of the Western Cape, Cape Town, South Africa

**Keywords:** Adolescent health, Young people, Sexual health, Reproductive health, Humanitarian, Conflict, Crises, Emergencies, Systematic review

## Abstract

**Background:**

Young people including adolescents face barriers to healthcare and increased risk of poor sexual and reproductive health (SRH), which are exacerbated in humanitarian settings. Our systematic review assessed the evidence on SRH interventions for young people including adolescents in humanitarian settings, strategies to increase their utilisation and their effects on health outcomes.

**Methods:**

We searched peer-reviewed and grey literature published between 1980 and 2018 using search terms for adolescents, young people, humanitarian crises in low- and middle- income countries and SRH in four databases and relevant websites. We analysed literature matching pre-defined inclusion criteria using narrative synthesis methodology, and appraised for study quality.

**Findings:**

We found nine peer-reviewed and five grey literature articles, the majority published post-2012 and mostly high- or medium-quality, focusing on prevention of unintended pregnancies, HIV/STIs, maternal and newborn health, and prevention of sexual and gender-based violence. We found no studies on prevention of mother-to-child transmission (PMTCT), safe abortion, post-abortion care, urogenital fistulae or female genital mutilation (FGM). Thirteen studies reported positive effects on outcomes (majority were positive changes in knowledge and attitudes), seven studies reported no effects in some SRH outcomes measured, and one study reported a decrease in number of new and repeat FP clients. Strategies to increase intervention utilisation by young people include adolescent-friendly spaces, peer workers, school-based activities, and involving young people.

**Discussion:**

Young people, including adolescents, continue to be a neglected group in humanitarian settings. While we found evidence that some SRH interventions for young people are being implemented, there are insufficient details of specific intervention components and outcome measurements to adequately map these interventions. Efforts to address this key population’s SRH needs and evaluate effective implementation modalities require urgent attention. Specifically, greater quantity and quality of evidence on programmatic implementation of these interventions are needed, especially for comprehensive abortion care, PMTCT, urogenital fistulae, FGM, and for LGBTQI populations and persons with disabilities. If embedded within a broader SRH programme, implementers and/or researchers should include young people-specific strategies, targeted at both girls/women and boys/men where appropriate, and collect age- and sex-disaggregated data to help ascertain if this population’s diverse needs are being addressed.

## Introduction

In 2018, the United Nations (UN) estimated that 135.7 million people were in need of humanitarian aid in 25 countries around the world [1]. The United Nations High Commissioner for Refugees (UNHCR) reports that this large population includes 70.8 million people who have been forcibly displaced from their homes because of conflict and persecution, including 25·9 million refugees, over half of whom are under the age of 18 [2]. These populations are more likely to have poor health outcomes, including in sexual and reproductive health (SRH), due to disrupted services, lack of health supplies, scarcity of trained health workers, and increased risk of sexual violence [3]. Young people in these settings will often find themselves in high-risk situations and may be forced to take on adult roles within their families and communities [4].

Adolescents are defined by the World Health Organization (WHO) as persons aged 10–19 years. However, research concerning adolescents is often extended to include persons aged 10–24 years, defined as ‘young people’ [5]. While young people are not a homogenous group, they are a vulnerable population group and over the past two decades, increased attention has been paid to addressing their unique health needs [6]. This population undergo an intense period of physical, cognitive, emotional and social development, setting them apart from children and adults [7]. This rapid development results in new behaviours, which can have an impact on short- and long-term health outcomes [8], requiring a tailored health care approach [7].

Despite the specific needs and vulnerabilities faced by young people, most services are not organised to recognise or meet these needs [7]. For example, young people often face barriers when accessing health care including a lack of knowledge about their health and health services, an inability to travel to access these services, restrictive laws and judgemental attitudes of health care workers [7, 9, 10]. Additionally, young people are in a period of their life with increased need for privacy and confidentiality with a greater fear of embarrassment and judgement by others [9]. They therefore require services that are respectful and responsive to their needs [9].

The vulnerability of young people in humanitarian settings is compounded. As families, communities and social groups are disrupted in these settings [5], adolescents may find themselves in high-risk situations and may be forced to take on adult roles within their families and communities [3]. There can also be interruption of adolescent SRH service delivery resulting in a lack of access to and information about available services and an increase in the risk of sexual exploitation and abuse [11]. It is for this reason that particular emphasis needs to be placed on making SRH services in humanitarian settings young people-inclusive and tailored towards their specific needs. At the International Conference on Population and Development (ICPD) in Cairo in 1994, the SRH rights of those living in crisis-affected settings were first recognised [12]. This led to the formation of the Inter-Agency Working Group (IAWG) for Reproductive Health in Refugee Settings and the development of the Inter-Agency Field Manual (IAFM) for Reproductive Health in Humanitarian Settings [13–15]. The IAFM introduces the Minimum Initial Services Package (MISP), which is a set of priority interventions (Panel 1) and actions designed to reduce morbidity and mortality and provide guidelines for coordinated SRH services in the early stages of an emergency as well as guidelines for comprehensive services once the situation has stabilised [13, 14].

The IAFM also includes a chapter on adolescent SRH which, along with the Adolescent Sexual and Reproductive Health Toolkit for Humanitarian Settings published by the United Nations Population Fund (UNFPA) [5], provides guidance on making SRH services in these settings inclusive to adolescents and young people.

Despite this guidance, there has been no systematic review focusing solely on SRH interventions for young people in humanitarian settings. A previous review on SRH interventions in humanitarian settings in the general population found four interventions that targeted adolescents, all of which were HIV prevention interventions [16]. Similarly, Warren et al (2015) found generally low-quality evidence on the effectiveness of SRH interventions in humanitarian settings and did not find any young people-focused studies [3]. Singh et al (2018) assessed the utilisation of SRH services in humanitarian crises and found only one study targeted to this population [17].

Young people are a key population in humanitarian settings, and yet little is known about the evaluation of SRH interventions directed to them. In order to address these gaps in knowledge, our systematic review aims to assess the evidence on the spectrum of SRH interventions being delivered to young people including adolescents in humanitarian settings, as well as the strategies to increase their utilisation and their effects on health outcomes.

## Methods

This systematic review follows the reporting guidelines as set out in the Preferred Reporting Items for Systematic Reviews and Meta-Analyses (PRISMA) statement [18]. Table 1 describes the inclusion and exclusion criteria.

**Table 1:** Inclusion and exclusion criteria for the systematic review

Category	Included	Excluded
Population of interest	Young people including adolescents (male and female) aged 10–24 years living in humanitarian settings in low- and middle-income countries (LMICs)	Populations living in humanitarian settings in high-income countries
Intervention	Any intervention aimed at improving SRH outcomes as defined in the Minimum Initial Service Package as part of the Inter-Agency Field Manual for Reproductive Health in Humanitarian Settings [13]	
Article type	Any quantitative or qualitative study describing an SRH intervention and measuring an SRH outcome.	Any study with no specific SRH intervention or that only describes needs, prevalence or risk factors.
Crisis type	Any acute or protracted armed conflict, disease outbreak, or natural disaster	Studies conducted before a crisis has occurred
Publication date	1980–2018	
Language	English, French	Other languages

We defined a humanitarian setting as one in which ‘an event or series of events has resulted in a critical threat to health, safety, security or well-being of a community or other large group of people’. [15] The affected community is no longer able to cope and external assistance, whether from the national or international level, is required. The event can be a natural or man-made disaster [15], and settings can range from acute to stabilised. While we recognise that some forcibly-displaced populations live in stable, high-income settings, we focused only on interventions implemented in low- and middle-income countries (LMIC), as the majority of humanitarian settings occur in LMIC and the resources available in high-income countries to deal with humanitarian emergencies are different and much greater than those in LMIC [3].

Both peer-reviewed and grey literature were included. All quantitative and qualitative research studies describing an SRH intervention for young people including adolescents and measuring an SRH outcome were included. Outcomes of interest were based on those outlined in the MISP as part of the IAWG Field Manual, focusing on measures of prevention of sexual violence, prevention of transmission and reduction of morbidity and mortality related to HIV and other STIs, prevention of excess maternal and newborn morbidity and mortality, prevention of unintended pregnancies, and safe abortion [13].

### Search strategy

Our search strategy focused on literature published between 1980 and 2018. It was adapted from previous systematic reviews on SRH in humanitarian settings [3, 17] and finalised with help from librarians trained in systematic review methodology.

The search included terms in the following categories: 1) humanitarian settings and crises, 2) LMIC, 3) SRH interventions, and 4) young people including adolescents. Both free-text searching and subject headings were used. The full search strategy can be found in the Additional file 1.

The search included peer-reviewed literature retrieved from the following databases: MEDLINE, EMBASE, Global Health and PsycINFO. A grey literature search was conducted using the following online resources and websites: Results for Development, Reproductive Health Response in Crisis Consortium, Médecins Sans Frontières, UNFPA, RAISE initiative, IAWG, Save the Children, The International Rescue Committee (IRC), CARE International, International Committee of the Red Cross, International Planned Parenthood Federation, AIDS Alliance, Marie Stopes International, Women’s Refugee Commission (WRC), Population Council and The Coalition for Adolescent Girls. Broad search terms such as ‘young’, ‘adolescent’, ‘humanitarian’ and ‘sexual health’ as well as topic-based searches were used. Advanced searches using similar terms were run on Google Scholar and the Popline database.

Reference lists of included peer-reviewed and grey literature publications and relevant systematic reviews were screened for additional articles. Experts in the field of adolescent SRH in humanitarian settings, including members of IAWG’s adolescent SRH sub-working group, were contacted to identify literature not found during the systematic search. Only literature from 1980 to 2018 was searched as a previous review found no SRH studies published prior to that date [19].

### Study selection and data extraction

**Fig. 1:**
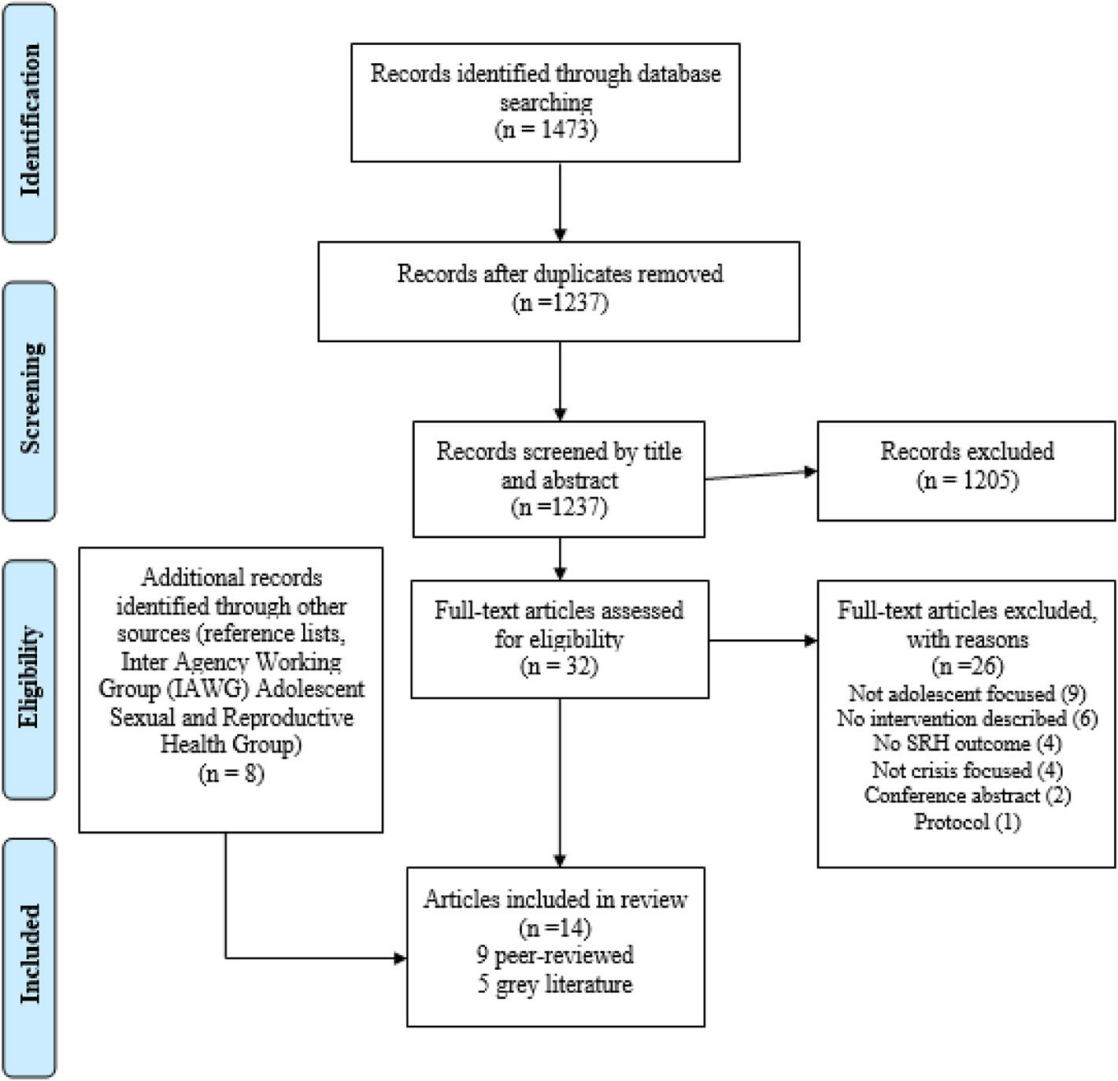
PRISMA flow-chart [17] for systematic review on sexual and reproductive health interventions for young people including adolescents in humanitarian crises settings

All citations from the database searches were exported to Mendeley referencing software and then to Excel. LJ and NSS conducted the screening independently (Fig. 1) using pre-defined inclusion and exclusion criteria (Table 1). Data from the included interventions were extracted under the following headings: author and year, study setting, target population, crisis type, SRH domain, study design, study outcomes, intervention description, results, implementing bodies and funders.

### Data analysis

Due to the heterogeneity of interventions, settings, and outcomes measured, we used a narrative synthesis approach to analyse findings [20]. Findings were reviewed and synthesised by the lead author (LJ) following a process of constant comparison. After drafting synthesised findings, authors (LJ, NSS) revisited the original articles to check their interpretations.

The quality of included articles was assessed using critical appraisal checklists appropriate to the type of publication, described in Table 2.

**Table 2:** Quality appraisal checklists used in the systematic review

Study design	Quality appraisal checklist
Randomised control trials (RCTs)	Consolidated Standard of Reporting Trials (CONSORT) checklist [21]
Observational study	Strengthening of Observational studies in Epidemiology (STROBE) checklist [22]
Before- and after-study	National Heart, Lung and Blood Institute’s checklist [23]
Qualitative study	Critical Appraisal Skills Programme (CASP) checklist [24]
Case study	Centre for Evidence-Based Medicine checklist [25]
Grey literature	Authority, accuracy, coverage, objectivity, date, significance (AACODS) checklist [26]

All articles were given a final quality score which was converted into a percentage of the total achievable score. Articles were then given a rating of low-, medium- or high-quality based on that percentage. Low-quality studies scored between 0 and 33%, medium-quality scored between 34 and 66% and high-quality studies scored 67% and above. These quality thresholds have been used in previous reviews of SRH interventions in humanitarian settings [3, 17].

## Results

### Overview of studies

We screened titles and abstracts of 1237 records from peer-reviewed databases and 8 articles from other sources. After full-text screening, 14 articles (i.e. 9 peer-reviewed studies [27–35] and 5 grey literature articles [36–40]) met our inclusion criteria and were included in the review (Fig. 1). Two grey literature articles described more than one SRH intervention for young people, and several interventions were described in more than one article, so we present findings from 15 individual SRH interventions for young people in humanitarian settings (Table 3). Of the 14 articles included in the review, 13 were published between 2012 and 2018, and one was published before 2012, in 2006 [27].

**Table 3:** Characteristics and key findings of studies assessing sexual and reproductive health outcomes for young people in humanitarian settings

Author (year) & Article type	Study design and quality	Study setting	Target population	Intervention (strategy, implementer, donor)	Key findings
Prevention of unintended pregnancies					
Nehme and Spilotros 2018 [40] Grey literature	Case Study Medium quality	DRC Urban Armed conflict (Protracted)	Adolescent girls (Age not specified)	Provision of family planning services through adolescent SRH training for providers and adolescent participatory workshops Implementer: International Rescue Committee (IRC) Donor: David and Lucille Packard Foundation	Number of adolescents accepting new methods of contraception increased from 67 in March 2017 to 156 in December 2017. 89·0% of adolescent clients accepted long-acting methodsUse of sexually transmitted infection (STI) services steadily increased.
Prevention of transmission of and reduction in morbidity and mortality due to HIV and sexually transmitted infections (STIs)					
Atwood et al 2012 [33] Peer-reviewed	Randomised control trial (RCT) Medium quality	Liberia Urban Armed conflict (Post-conflict)	General In-school 6th grade youth (Age not specified)	Eight module curriculum delivered in-school over 8 weeks by health educators Implementer: Not specified Donor: National Institute of Mental Health	Significantly increased protective peer norms (*p* < 0·05) and positive attitudes towards condoms (*p* < 0·05). Increased frequency of condom use at 9 months. No significant improvement in age of sexual initiation and number of multiple sex partnerships.
Atwood et al 2012 [32] Peer-reviewed	RCT Medium quality	Liberia Urban Armed conflict (Post-conflict)	General In-school 6th grade youth engaging in transactional sex Ages 14–18	Eight module curriculum delivered in-school over 8 weeks by health educators Implementer: Not specified Donor: National Institute of Mental Health	No impact on risk factors for adolescents that engaged in transactional sex For participants comprised of adolescents engaging in transactional sex for a range of rewards, the intervention group was found to have a higher number of sex partners (β = 0·33, p < 0·01) and increased frequency of sex (β = 0·27, p < 0·01) in the previous three months.
Casey et al 2006 [27] Peer-reviewed	Controlled before-and-after Medium quality	Sierra Leone Urban Armed conflict (Protracted)	General Ages 15–24	Intensive outreach education by peers, including a focus on improving negotiation skills. Free condom distribution. Implementer: American Refugee Committee International Donor: David and Lucille Packard Foundation	Increase in ability to name 3 HIV prevention methods in male (from 4·0% to 45·0%) and females (from 4·0% to 36·0%), both p < 0·01. Reported condom use at last sex increased: Males 16·0% to 37·0%, females 16·0% to 46·0%.
Talbot 2013 [30] Peer-reviewed	One-arm cohort High quality	Rwanda Peri-Urban Armed conflict (Post-conflict)	General Rwandan orphans Ages 15–25	Incorporating HIV prevention education activities into an existing mental health intervention. Implementer: Uyisenga N’Mzanzi Donor: Gilead Foundation	Reported condom use when engaging in high-risk behaviour increased from 54·0% at baseline to 78·0% at study completion. Percentage reporting exchanging sex for money, food or favours decreased from 13·0% to 9·0% (p = 0·006). HIV risk-taking behaviour assessed via the AIDS Clinical Trials Group (ACTG) Sexual Behaviour Index did not change significantly over the 12-month study period (23·0% (0·42) at baseline vs. 24·0% (0·43) at 12 months, *p* = 0·858).
Prevention of sexual and gender-based violence and response to the needs of survivors					
Lilleston et al (2018) [31] Peer-reviewed	Qualitative High quality	Lebanon Rural Armed conflict (Protracted)	Refugees Adolescent girls (Age not specified)	GBV mobile service delivery designed to complement static GBV services.Each mobile team included an adolescent girls’ assistant. Implementer: IRC Donor: US Department of State, NoVo Foundation, Swedish International Development Corporation	Adolescents developed trusting friendships as a result of the service, received emotional support from the adolescent assistant, improved family relationships, felt safer and more confident, and had increased knowledge and skills to feel safer when leaving the house.
Stark 2018 [28] Peer-reviewed	RCT High quality	Ethiopia Refugee camps Armed conflict (Protracted)	Refugees Adolescent girls Ages 13–19	40 fixed-curriculum, mentor-facilitated sessions once a week for 10 months.Caregivers also participated in monthly discussion sessions. Implementer: IRC Donor: Department for International Development (DFID)	Intervention group no more or less likely to engage in transactional sexual exploitation compared to control group after receiving the interventionOR = 0·62 (95% CI 0·3–1·26) p = 0·082
Tanner et al 2017 [39] Grey literature	DRC: two-arm waitlisted RCTEthiopia: two-group waitlisted cluster RCTPakistan: single group pre- and post-test survey evaluationAll contained qualitative component High quality	DRC Ethiopia Pakistan Refugee camps Armed conflict (Protracted)	General/refugees Adolescent girls DRC: age 10–14 Ethiopia: age 13–19 Pakistan: age 10–19	40 fixed-curriculum, mentor-facilitated sessions once a week for 10 months.Caregivers also participated in monthly discussion sessions. Implementer: IRC, Colombia University Donor: DFID	Girls participating in the programme reported having greater support networks and a safe place to interact with other adolescent girls. Girls in Ethiopia were nearly twice as likely to report having friends and the girls in DRC who reported having four or more friends increased from 54% at baseline to 96% at endline Girls participating in the intervention were also found to have greater hope for the future. Girls in the intervention had twice the odds of reporting girls should be 18 or older when they have their first child and almost twice the odds of reporting girls should be 18 or older when they get married. Improved knowledge of SGBV services among girls and the intervention made services more adolescent friendly. Caregivers reported greater warmth and affection and lower overall rejection of their daughters. No significant changes in exposure to SGBV or the attitudes of girls towards gender and SGBV. No effect on levels of violence experienced. The intervention was found to be feasible and acceptable
Intervention targeting outcomes across multiple SRH domains					
Barnett et al (2018) [34] Peer-reviewed	Case Study Medium quality	Sierra Leone Rural Disease outbreak (Acute and stabilised)	General Ages 12–18	Radio educational programmes aimed at increasing awareness of teen pregnancy, sexual violence, HIV and hygiene practices. Implementer: Child-to-Child, Pikin-to-Pikin Donor: The Circle	Reached an audience of 136,678 listeners. Children showed good recall of key messages such as how to prevent teenage pregnancy. Children talked to their peers about what they had learnt, thus spreading the message. Teachers had increased knowledge about sensitive topics such as teenage pregnancy and confidently talked to children about these.
Bosmans et al 2012 [29] Peer-reviewed	Qualitative Low quality	Colombia Internally displaced persons’ (IDPs) settlements Armed conflict (Protracted)	Adolescent IDPs (Age not specified)	Use of different forms of interactive theatre and arts in workshops to address taboos surrounding SRH issues and approach topics such as disease, domestic violence, sexual abuse, unwanted pregnancy, friendship, joy and love.Used to transmit and procure SRH information at schools attended by IDP adolescents.Training for public health staff in medical aspects of adolescent sexual and reproductive health (ASRH) and adolescent-friendly services. Implementer: Colombian authorities, United Nations (UN) agencies, various Non-governmental organisation (NGOs) (not specified) and local universities Donor: United Nations Population Fund (UNFPA) and Government of Belgium	Intervention restored dignity and increased awareness of rights. One participant noted that the use of theatre and dance “created trust and opened a space in which these matters could be discussed” [29]. Improved awareness of and attitudes to SRH among adolescents, caregivers and health workers. Availability of and access to services remained a problem.
Chaudhary et al (2017) Peer-reviewed [35]	Case study High quality	Nepal Rural Natural disaster (Acute)	Adolescent girls and boys (Age not specified)	Establishment of an ASRH working group. Adolescent-friendly service corners set up in reproductive health camps, run by trained adolescent facilitators and volunteers. Establishment of linkages between adolescent-friendly corners and adolescent-friendly services in health facilities. Implementer: Nepal Ministry of Health, UNFPA, World Health Organization (WHO) Donor: Not specified	Adolescent-friendly corners served over 4231 young people. More than 14,666 adolescents received ASRH services overall.
Tanabe et al 2012 [38] Grey literature	Case study High quality	Thailand Peri-urban Armed conflict (Protracted)	Migrant adolescents Ages 15–24	Youth centre- based workshops covering: reproductive anatomy, physical and emotional changes during adolescence, FP, sex and gender, HIV/STI transmission and prevention, consequences of unsafe abortion. Implementer: Adolescent Reproductive Health Network Donor: Not specified	Numbers of new and repeat FP clients decreased form May–June 2011 to May–June 2012. NB as the intervention was been operational since 2008, it is not clear whether these data are representative of contraceptive uptake since initiation of the intervention.
		Colombia Semi-rural Armed conflict (Protracted)	IDPs Ages 10–24	Clinics, mobile health brigades and community education on SRH (content not specified). Training of youth peer educators. Adolescent participation in development of training materials. Implementer: Profamilia, The Regional Human Rights Commission (RHRC) Donor: Not specified	Percentage of young women in a relationship using a modern contraceptive method increased from 47·0% to 54·8% (ages 15–19) and 61·7 to 71·7% (ages 20–24) from 2008 to 2010. Percentage of young women not in a relationship using a method increased for injection and IUD (except for IUD in ages 20–24). Large increase in implant use for ages 20–24. No implant uptake in younger age groups.
		Uganda Peri-urban Armed conflict (Stabilised)	Previous IDPs, now returnees Ages 10–24	Consultations at youth centre, community outreach, school visits, home visits, support groups, clubs, edutainment, radio and newspaper. Services include: STI diagnosis and treatment, HIV counselling and testing, FP, male circumcision, post-rape care. Implementer: Straight Talk Foundation Donor: Not specified	From 2007 to 2012: Initial increase in number of clients accepting pill, injection and condoms but all returned to baseline by 2010. Initial increase in total FP clients and total clients accepting a method but also returned to baseline
Tanabe et al 2013 [37] Grey literature	Case study High quality	DRC Setting not specified Armed conflict (Protracted)	Adolescents Age 10–19	Training of medical staff on ASRH toolkit. Support groups for pregnant adolescents. Peer-educator led activities for in-school adolescents including HIV/STI prevention, pregnancy prevention, and gender norms. Implementer: Save the Children, Women’s Refugee Commission (WRC), The UN Refugee Agency (UNHCR) Donor:	Improvement in adolescent friendly services checklists. Increased use of SRH services (mainly ages 15–19). Increased knowledge among pregnant adolescents (not significant). Improvements seen in attitudes of in-school adolescents (p < 0·05). A four-fold increase in the number of adolescents aged 15–19 years attending the adolescent counselling room at the health centre from 14 adolescents in August 2013 to 56 adolescents in October 2013. There was a drop-off in November 2013 with only 22 adolescents attending the counselling room. However, as this is the final data point, it is not known if this was a new downward trend. Facility staff attributed this finding to adolescents feeling comfortable directly accessing health services without first attending the adolescent counselling room.
UNFPA 2016 [36] Grey literature	Case study Medium quality	Malawi Camps Natural disaster (Acute)	IDP adolescents (Age not specified)	Youth clubs in displacement camps providing integrated STI/ HIV, SGBV, and young people’s welfare within MISP interventions Implementer: UNFPA, Youth Net and Counseling (YONECO), Centre for Victimised Women and Children (CAYWOC) Donor:	Established 32 youth clubs, reached more than 2000 adolescents and young people The adolescent involvement was reported to increase the sustainability of the clubs as well as the buy-in from the community. It was also reported that in Nepal following an earthquake the involvement of adolescents in the emergency response allowed the interventions to impact previously difficult to reach groups of adolescents.
	Case study Medium quality	Nepal Rural Natural disaster (Acute)	IDP adolescents (Age not specified)	Reproductive health camps included a separate tent set up specifically for adolescents. Young volunteers trained on a wide range of SRH issues including menstrual hygiene, STI/HIV prevention, child marriage and adolescent pregnancy, delivery and childbirth, and SGBV. Implementation of the MISP. Implementer: UNFPA, Manmohan Memorial Community Hospital (MMMCH), Pharping, ADRA Nepal Donor: Not specified	Established 132 mobile reproductive health camps, reached 16,977 adolescents
	Case study Medium quality	Philippines Camps Natural disaster (Acute)	General/IDP adolescents (Age not specified)	Young people recruited as volunteers and active partners. Volunteers trained to conduct awareness-raising sessions on safe motherhood, FP, STIs, and SGBV. Implementation of the MISP. Implementer: UNFPA, Family Planning Organisation of the Philippines (FPOP), Y-PEER, Pilipinas, Department of Health Donor: Not specified	Trained 30 youth volunteers. Distributed hygiene kits and provided services for 3967 young people

### Study design and quality

The nine peer-reviewed articles included three randomised controlled trials (RCTs) [28, 32, 33], one cohort study [30], two case studies [34, 35], one before-and-after study [27], and two qualitative studies [29, 31] (Table 3). Of the grey literature articles (*n* = 5), four were case studies [37–40] and one presented two RCTs and a before-and-after study [39].

Among the peer-reviewed literature, all three RCTs were considered to be medium-quality [28, 32, 33] (Table 3), as none of them adequately described sample size calculation or the randomisation process. The observational study [30] included was high-quality despite inadequate consideration of confounders in the analysis. Of the two qualitative studies, one was high-quality and the other of low-quality. The three remaining peer-reviewed articles were found to be medium-quality [27, 34, 35]. Among the grey literature articles, three were found to be high-quality [37–39], while two were considered medium-quality [36, 40].

### Study setting

One intervention was delivered in an acute disease outbreak setting, and three in natural disaster settings (Table 3). The remaining 11 interventions were delivered in areas affected by armed conflict, either in protracted violence or post-conflict settings. Nine interventions were implemented in Sub-Saharan Africa (SSA), with one of these interventions also being implemented in Pakistan. Three interventions were implemented only in Asia, while the remaining interventions were implemented in South America (*n* = 2) and the Middle East (*n* = 1).

### Target population

All interventions targeted adolescents, with five extending the eligible age group to include young people [27, 30, 38] (Table 3). Only four interventions were explicitly inclusive of very young adolescents aged 10–14 years [37–39]. Seven interventions did not specify an age-range but mentioned targeting adolescents. Nine interventions targeted both males and females though only one study provided differentiated results [27]. While all interventions were inclusive of girls or young women, there were no interventions targeted exclusively at adolescent boys or young men.

### Overview of SRH interventions

#### SRH domains covered by the interventions

Over half (*n* = 9) of the 15 interventions included in the review provided more than one element of SRH as part of their interventions to reach adolescents and young people in humanitarian settings (Table 3). The included studies focused on prevention of unintended pregnancies (*n* = 8), prevention of the transmission of and morbidity and mortality related to HIV and STIs (n = 8), prevention of excess maternal and newborn morbidity and mortality (*n* = 4), and prevention of sexual and gender-based violence (SGBV) (*n* = 2). We identified no studies focused on prevention of mother-to-child transmission (PMTCT), safe abortion, post-abortion care, urogenital fistulae or female genital mutilation (FGM). Furthermore, we found no studies targeting LGTBQI populations or young people with disabilities.

### Strategies to increase utilisation of SRH services

#### Adolescent-friendly spaces

Eight interventions described having adolescent- or youth-friendly services as part of their intervention [29, 36–38] (Table 3). However, adolescent-friendly services were not always defined and it is therefore difficult to determine if this was actually achieved. Four case studies utilised an adapted version of the Inter-Agency Field Manual Checklist (Table 4) to assess if an intervention was adolescent-friendly, and were found to fulfil the majority of the criteria [37, 38].

**Table 4:** Adapted Version of the Inter-Agency Field Manual Checklist for Adolescent Friendly Services [37, 38]

Adolescent-Friendly Checklist	
Health Facility	• Convenient hours • Convenient location • Adequate space and sufficient privacy • Comfortable surroundings
Provider	• Respect for adolescents • Non-judgmental attitude • Privacy and confidentiality honoured • Peer counselling available • Same-sex providers when possible • Strict confidentiality maintained • Staff trained in youth-friendly health service characteristics
Administrative	• Adolescent involvement • Boys and young men welcome • Necessary referrals available • Affordable fees • Drop-in clients welcome • Publicity and recruitment that informs and reassures adolescents

#### Peer workers

Casey et al (2006) described an intervention in a protracted armed conflict setting in Sierra Leone targeting male and female young people and provided intensive peer outreach education in order to improve condom negotiating skills, and free distribution of male condoms by peer workers [27].

Tanabe et al (2013) described using peer educators to conduct outreach and sensitisation to SRH issues including but not limited to prevention of unintended pregnancies, and prevention of HIV and STIS, in the community [38]. In Colombia, Tanabe et al (2012) used peer workers to increase community support for SRH interventions for young people and target vulnerable populations who might otherwise have difficulty accessing services [38]. In Thailand, Tanabe et al. (2012) also used peer educators to educate migrant youth on SRH issues [38].

The Creating Opportunities through Mentoring, Parental Involvement and Safe Spaces (COMPASS) intervention implemented in the DRC, Ethiopia and Pakistan focused on prevention of SGBV by using older girls aged 18–30 to act as mentors and facilitate a fixed-curriculum programme targeted to adolescent girls and their parents or caregivers, with an aim of reducing SGBV in girls aged 10–19 [28, 39]. The mentors came from a similar background to the participants and in Pakistan, they were former participants of the programme.

Using peers as part of an intervention did present some challenges. All three case studies by Tanabe et al (2012) reported that retention of peer workers is a challenge, especially as they often work as volunteers and lack incentive to continue, which can affect the sustainability of the intervention [38]. A concern arising from the COMPASS intervention was that, as peer workers were from the same background as adolescents participating in the programme, they might reinforce harmful societal norms regarding gender roles and gender-based violence [39].

#### Involving adolescents

IRC piloted an intervention in the DRC designed to increase uptake of contraception among adolescent girls through a participatory approach [40]. Adolescent girls were included at all stages of implementing the intervention, from workshops aimed at identifying and setting priorities for adolescent SRH services, to forming coordination committees for the implementation of the decided-upon actions. They also participated in progress monitoring and supervision visits to facilities. However, they did not participate in the overall research process.

In response to floods in Malawi, adolescents were involved in planning and implementing youth clubs within displacement camps. These youth clubs aimed to provide a range of SRH services in an integrated approach to adolescent girls and boys [36].A youth centre in Uganda also used adolescent input in its design and employed community outreach and home visits to deliver SRH care [38].

#### Engaging communities

Tanabe et al (2012)’s case studies from Thailand, Uganda and Colombia reported initial resistance from adults in the community to the provision of SRH services to adolescents, mostly expressed through a fear that SRH activities might encourage young people to engage in sexual activity [38]. The three interventions reported successfully overcoming this resistance by building trust with the community through engagement of community leaders [38].

#### School-based activities

The Making Proud Choices intervention in post-conflict Liberia targeted in-school 6th grade youth and provided an eight module training programme delivered in school by health educators, which aimed to prevent HIV by promoting positive attitudes towards condom use and increasing skills required to negotiate safer sex practices [32, 33].

A case study from the DRC targeted in-school adolescents aged 12–14 years [37]. Most of the activities occurred within the schools and involved the teachers being trained to “champion” adolescent SRH [37]. Peer educators were selected by their classmates and trained on various SRH topics including HIV and STI prevention, menstruation, and puberty. Peer education sessions were mainly conducted outside of normal school hours, however, many in-school adolescents were unable to attend as they were expected to be home immediately after school, demonstrating the need for school-based activities to be conducted within regular school hours [37].

#### Mobile clinics

Lilleston et al. (2018) delivered a mobile SGBV service targeting Syrian refugee women in Lebanon [31]. Three mobile teams each consisted of a community mobiliser, a caseworker, and an adolescent girls’ assistant. All team members were women, except for one male community mobiliser who rotated between the three teams [31]. Each mobile team also included an adolescent girls’ assistant, whom the girls said provided them with emotional support. This service aimed to complement existing static services and provided activities that addressed psychosocial issues and risk mitigation as well as individual case management.

#### Integration with non-SRH services

In Rwanda, the Uyisenga N’Manzi intervention aimed to reduce HIV risk amongst its participants by integrating HIV prevention education into an existing mental health intervention. This intervention included group discussions, lectures and presentations from staff covering sexuality, HIV and prevention strategies [30].

#### Additional strategies to increase utilisation of SRH services

In Colombia, Bosmans et al (2012) used the arts as a form of health education [29]. Interactive theatre, dancing, painting and singing were used to promote body awareness and address SRH issues, including taboo topics, among internally displaced youth [29].

In Sierra Leone, Barnett et al. (2018) adapted pre-existing radio programme to continue to educate youth when schools shut down during a crisis [34]. This included a radio programme aimed at 12–18 year olds that aimed to increase awareness of prevention of teen pregnancy, HIV and violence [34].

### Implementers and donors of SRH interventions

Over half (*n* = 9) of the 15 SRH interventions targeted to young people in this review were implemented by large international agencies e.g. UNFPA, IRC, WRC, ARC International, WHO and Save the Children, usually in collaboration with local authorities and organisations. Information on donors for SRH interventions in humanitarian settings was only reported for 8 of the 15 interventions, and included the David and Lucille Packard Foundation [27, 40], the Department for International Development (DFID) [28, 39], the National Institute of Mental Health [32, 33], Gilead [30], and the Government of Belgium [29]. One intervention was funded as a collaboration between the US Department of State, NoVo Foundation and the Swedish International Development Cooperation [31].

### Key findings

Key findings from each study are presented in Table 3. The majority of studies measured output data (e.g. number of services provided, utilisation rates), and changes in knowledge and attitudes, whereas fewer studies measured changes in behaviour or risk. Nearly all studies (*n* = 13) reported some positive SRH outcomes, the majority of which were positive changes in knowledge and attitudes, half reported no effects in some SRH outcomes measured (*n* = 7), and one study reported a decrease in number of new and repeat contraceptive clients.

## Discussion

This is the first systematic review to assess the evidence base on SRH interventions with health outcome data for young people, including adolescents, in humanitarian settings. All but one of the 14 included articles were published in the previous 7 years, showing that studies on SRH interventions for young people in humanitarian settings have only recently started to become a priority, which is consistent with global trends related to research and programming on health services for young people [9].

The review included a range of study designs with mostly high to medium quality, which is consistent with previous reviews on SRH interventions in humanitarian settings [3, 17]. While the quality of the articles could be assessed through the use of critical appraisal tools, we were unable to determine the quality of individual SRH interventions. This is because, while programme reporting standards exist [41], these were not adhered to in the included articles.

There are known challenges to conducting research in humanitarian settings. These challenges include security risks for researchers, lack of resources to conduct research and instability and mobility of the study population [3]. However, a previous review of health care services in humanitarian settings found high-quality studies in the fields of mental health and communicable diseases which suggests that it is possible for good research to be done in these settings [19]. Another barrier to conducting research may be the special ethical considerations that need to be made when working with young people and SRH, as described by WHO [42]. Legal requirements may require parental consent for participation in research, especially for younger adolescents, which may be a challenge for recruitment in humanitarian settings, where young people are often separated from their parents.

This systematic review found few studies of SRH interventions for young people in humanitarian settings, similar to that found in previous reviews of SRH interventions in humanitarian settings [3, 17]. Our findings show that while there has been an increase in conducting research and documenting programming in this field in recent years, we need better quantity and quality of data using rigorous evaluation methods on SRH interventions for young people and adolescents. These data should be collected and analysed via standardised monitoring systems, with integration between humanitarian and local health information systems where possible and appropriate. It is important that when SRH data are collected, they are disaggregated by age using standard age groups in order to understand gaps in SRH service provision for this population in humanitarian settings and to make evidence-based decisions when allocating financial resources.

Over half (*n* = 9) of the interventions included in this review were described in the grey literature and all of these were from reports produced by large international non-governmental organisations (NGOs). The interventions included may therefore not be a good representation of all SRH interventions for young people in humanitarian settings but rather reflect the main funders and donors active in this field. It may be the case that those interventions implemented by local governments and smaller organisations are less likely to be documented, evaluated and published due to capacity and resource constraints. NGOs may not always prioritise the publication of programme results, especially in peer-reviewed literature. The lack of studies in this field could therefore be due to challenges related to the setting and population, which have resulted in rigorous programme evaluation not being the norm in these settings, or due to a lack of funding for this type of research.

The interventions included in our review were implemented in acute disease outbreak (*n* = 1), natural disaster (*n* = 3), protracted armed conflict (*n* = 8) and post-conflict (n = 3) and settings in LMIC. Interventions focused on prevention of unintended pregnancies, HIV and STIs, maternal and newborn health, and prevention of SGBV, with an emphasis on reproductive ‘problems’ i.e. pregnancy and HIV, and little focus on sexual health including comprehensive sexuality education. We found no studies focusing on PMTCT, urogenital fistulae or FGM. Consistent with previous reviews assessing SRH in humanitarian settings, we found no studies documenting the provision of safe abortion services and few studies documenting post-abortion care services being delivered [3, 17]. None of the studies reported inclusion of young people from sexual minorities or those with disabilities. Strategies to increase utilisation of SRH interventions by young people include adolescent-friendly spaces, peer workers, school-based activities, and involving young people in the development, implementation and evaluation of interventions. Most of the positive SRH outcomes reported in the review showed an improvement in knowledge and attitudes, although these effects did not seem to always translate to changes in behaviour or risk status. This may be due to the short duration of implementation of the interventions, in most cases less than 12 months, which is likely insufficient to observe significant behavioural change. We found no data on economic evaluation of SRH interventions for young people in humanitarian crises settings.

The MISP is only intended to be implemented at the onset of an acute crises, and is meant to set the stage for a transition to comprehensive SRH services as the crisis stabilises [43]. These findings show that the limited spectrum of SRH services delivered in humanitarian settings has not changed since previous evaluations [16, 17]. Even though the MISP has been criticised by some actors as being too comprehensive to deliver at the start of a crisis [44], we would argue that the interventions described in this package should at the very least be provided to populations including young people in stabilised post-conflict settings.

None of the included interventions targeted boys only and, of the interventions aimed at reducing SGBV, all targeted only girls. However, research has shown that interventions that attempt to have a more gender transformative approach including targeting both men and women are more effective, especially when it comes to influencing long-term changes in societal norms and changing gender power relations [45]. In addition, very few interventions were explicitly inclusive of very young adolescents, indicating that this group, whose needs vary from older adolescents, are not being prioritised.

Of the interventions aiming to decrease the risk of SGBV, only the COMPASS intervention provided any outcome data, which showed no change in exposure to SGBV. This may be because end line data was collected directly following the 10-month intervention which may not have been a long enough time period to see real societal change resulting in decreased SGBV. Therefore, consideration needs to be given on how to assess the effectiveness of interventions like these targeting community-level change in an unstable and rapidly changing population.

Several interventions reported initial community resistance to the provision of SRH services to young people. Adults have been shown to influence the health-seeking behaviour of young people and, as such, it is essential that any new interventions have the support of the adult community and are seen as beneficial to this population [46]. It is also encouraging that many of interventions in this review reported involvement of young people at some point in the intervention cycle. Evidence has shown that meaningful engagement with and participation from young people increases positive outcomes of this population using health services [47].

There are a number of limitations to this systematic review. Firstly, due to the lack of published information on this topic, it is difficult to draw definitive conclusions. While the inclusion of grey literature expanded the evidence available for the review, it may have biased the types of interventions included as larger humanitarian organisations are more likely have the resources to write and publish reports. Therefore, it is important to note that what has been published may not represent which SRH interventions are actually being implemented for young people in humanitarian settings. Additionally, some implementing agencies prioritise research and publications more than others, and seek funding and partnerships specifically for research, e.g. this review includes four peer-reviewed articles (describing three interventions) with collaborators from academic institutions.

While we were able to assess the spectrum of SRH interventions delivered to young people, as well as their effectiveness, utilisation, and implementation modalities to a certain extent, limited data in the included studies did not allow us to assess distal determinants including relevant national, institutional, political, legal, and cultural factors which likely influenced the uptake and impact of these SRH interventions.

Additionally, we may have excluded relevant studies due to only including English and French language articles based on the study team’s capacity. We also excluded a number of articles, particularly in the grey literature, due to inadequate intervention descriptions and a lack of data on utilisation rate or health outcomes [38, 48, 49].

## Conclusions

This review highlights the need for a higher quantity and quality of studies documenting interventions addressing the comprehensive sexual and reproductive health needs of young people in their diversity in a range of humanitarian settings, including in acute and protracted conflict and natural disaster areas. While there is evidence that some SRH interventions for young people are being implemented, there are insufficient details of specific intervention components and outcome measurements to be able to adequately describe and assess these interventions. Implementers are using a number of different strategies including peer workers, adolescent-friendly settings and participatory approaches to increase utilisation of services by young people in humanitarian settings. However, there is a lack of research evaluating these interventions, which has implications for scale-up and sustainability. Interventions implemented in humanitarian settings in the future must invest in better documentation and evaluation.

## Supplementary information

**Additional file 1.** Search terms for systematic review of sexual and reproductive health interventions for young people including adolescents in humanitarian settings.

## Data Availability

All data generated or analysed during this study are included in this published article.

## Abbreviations

ASRH: Adolescent sexual and reproductive health
FGM: Female genital mutilation
IAFM: Inter-Agency Field Manual
IAWG: Inter-Agency Working Group
ICPD: International Conference on Population and Development
IRC: International Rescue Committee
LMIC: Low- and middle-income countries
MISP: Minimum Initial Services Package
PMTCT: Prevention of mother-to-child transmission
PRISMA: Preferred Reporting Items for Systematic Reviews and Meta-Analyses
RCT: Randomised controlled trial
SGBV: Sexual and gender-based violence
SRH: Sexual and reproductive health
STI: Sexually transmitted infection
UNFPA: United Nations Population Fund
UNHCR: United Nations High Commissioner for Refugees
WHO: World Health Organization
WRC: Women’s Refugee Commission
