# Supplementary material for: A forgotten group during humanitarian crises: a systematic review of sexual and reproductive health interventions for young people including adolescents in humanitarian settings
Source: Confl Health. 2019 Nov 27;13:57. doi: 10.1186/s13031-019-0240-y (PMC6880589; doi:10.1186/s13031-019-0240-y)
Supplement: Supplementary file 1 — Additional file 1. Search terms for systematic review of sexual and reproductive health interventions for young people including adolescents in humanitarian settings. [file 13031_2019_240_MOESM1_ESM.docx]

**Appendix 1**

**Search terms for systematic review of sexual and reproductive health interventions for young people including adolescents in humanitarian settings**

1. exp disasters/ [MeSH]
2. Disaster medicine/ [MeSH]
3. (humanitarian adj2 (crisis or crises or relief or response or agenc$)).tw.
4. humanitarian.tw.
5. (disaster adj3 (relief or plan$)).tw.
6. ((relief or aid) adj2 work$).tw.
7. Refugees/ [MeSH]
8. (refugee or evacuee or evacuated).tw.
9. (displace$ adj2 (force$ or population or human or internal$)).tw.
10. (internally displaced adj2 (person or people)).ti,ab.
11. exp Warfare/ [MeSH]
12. war.tw.
13. ((armed or zone) adj2 conflict$).tw.
14. (conflict affected adj3 (population$ or person$ or communit$)).tw.
15. Avalanches/ [MeSH]
16. Earthquakes/ [MeSH]
17. Floods/ [MeSH]
18. Landslides/ [MeSH]
19. Tidal Waves/ [MeSH]
20. Tsunamis/ [MeSH]
21. Cyclonic Storms/ [MeSH]
22. (typhoon$ or hurricane$ or cyclone$).tw.
23. (avalanche$ or earthquake$ or flood or floods or flooding or flooded or landslide$ or tsunami$).tw.
24. (disaster adj2 (natural or victim)).tw.
25. Droughts/ [MeSH]
26. drought$.tw.
27. Starvation/ [MeSH]
28. (starvation or famine$).tw.
29. or/1-28
30. Developing Countries/ [MeSH]
31. exp Asia/ [MeSH]
32. exp Africa/ [MeSH]
33. exp Pacific Islands/ [MeSH]
34. exp Eastern Europe/ [MeSH]
35. exp China/ [MeSH]
36. Balkan Peninsula/ or Europe eastern/
37. Caribbean region/ or Central America/ or “Gulf of Mexico”/ or Latin America/ or South America/
38. Atlantic Islands/ or Indian ocean islands/ or Macau/ or Pacific Islands/ or Philippines/ or Prince Edward Island/ or Svalbard/ or West Indies/
39. or/30-38
40. Japan/
41. 39 not 40

***Sexual and Reproductive Health***

1. Reproductive Health/ [MeSH]
2. exp Reproductive Medicine/ [MeSH]
3. Sexology/ [MeSH]
4. Sex Education/ [MeSH]
5. exp Sexual Behavior/ [MeSH]
6. exp Sexuality/ [MeSH]
7. Sexual Partners/ [MeSH]
8. “Sexual and Gender Minorities”/ [MeSH]
9. Sex Workers/ [MeSH]
10. Sexual Health/ [MeSH]
11. sexual health.ti,ab.
12. sexuality.ti,ab.
13. sex* education.ti,ab.
14. sexology.ti,ab.
15. sex counsel?ing.ti,ab.
16. sexual behavio?r.ti,ab.
17. sexual partner*.ti,ab.
18. sexual minorit*.ti,ab.
19. Minimum Initial Service Package.ti,ab.
20. Or/42-60

***Family Planning/Contraception***

1. reproductive plan*.ti,ab.
2. contracepti*.ti,ab.
3. birth control.ti,ab.
4. condom*.ti,ab.
5. “the pill”.ti,ab.
6. oral contraceptive.ti,ab.
7. microbicide.ti,ab.
8. diaphragm.ti,ab.
9. IUD.ti,ab.
10. Intrauterine device.ti,ab.
11. exp Contraceptive Devices/ [MeSH]
12. Levonorgestrel/ [MeSH]
13. Norethindrone/ [MeSH]
14. contraceptive implant*.ti,ab.
15. progestogen only contraceptive*.ti,ab.
16. progestogen implant*.ti,ab.
17. etonogestrel implant*.ti,ab.
18. Implanon.ti,ab.
19. subdermal contraceptive implant*.ti,ab.
20. Norplant.ti,ab.
21. Jadelle.ti,ab.
22. Sino-implant.ti,ab.
23. Depo Provera.ti,ab.
24. Nexplanon.ti,ab.
25. Norprogesterones.ti,ab.
26. natural family planning.ti,ab.
27. lactational amenorrhea.ti,ab.
28. LAM.ti,ab.
29. ((postpartum or post-partum) adj3 amenorrhea).ti,ab.
30. periodic abstinence.ti,ab.
31. rhythm method.ti,ab.
32. calendar method.ti,ab.
33. sexual abstinence.ti,ab.
34. exp Contraception/ [MeSH]
35. family planning.ti,ab.
36. Population Control/ [MeSH]
37. Contraceptives, oral/ [MeSH]
38. or/62-98
39. NOT Animals/ [MeSH]
40. NOT Animal experimentation/ [MeSH]
41. NOT Models, Animal/ [MeSH]
42. or/100-102
43. 99 not 103

***Medical abortion***

1. Abortion, Induced/ [MeSH]
2. Abortion, Incomplete/ [MeSH]
3. Abortion, Spontaneous/ [MeSH]
4. abortion.ti,ab.
5. miscarriage.ti,ab.
6. (pregnancy adj3 termination).ti,ab.
7. abortal.ti,ab.
8. (postabort* or post-abort*).ti,ab.
9. ((post-abortion or postabortion) and care).ti,ab.
10. incomplete abortion*.ti,ab.
11. (Mifepristone or RU486 or mifegyne).ti,ab.
12. (misoprostol or cytotec).ti,ab.
13. Medabon.ti,ab.
14. medication abortion.ti,ab.
15. medical abortion.ti,ab.
16. unsafe abortion*.ti,ab.
17. or/105-120

***Surgical abortion***

1. Extraction, Obstetrical/ [MeSH]
2. exp “Dilatation and Curettage”/ [MeSH]
3. surgical abortion.ti,ab.
4. (dilation and evacuation).ti,ab.
5. D&E.ti,ab.
6. suction curettage.ti,ab.
7. vacuum aspiration.ti,ab.
8. D&C.ti,ab.
9. menstrual regulation.ti,ab.
10. or/122-130

***Abortion-related complications***

1. Uterine Hemorrhage/ [MeSH]
2. Postpartum Hemorrhage/ [MeSH]
3. exp Pelvic Infection/ [MeSH]
4. exp Uterine Rupture/ [MeSH]
5. Pregnancy Complications/ [MeSH]
6. Abortion, Septic/ [MeSH]
7. endometritis.ti,ab.
8. parametritis.ti,ab.
9. metritis.ti,ab.
10. pelvic infection.ti,ab.
11. uterine infection.ti,ab.
12. uterine perforation.ti,ab.
13. abortion-related complications.ti,ab.
14. ectopic pregnancy.ti,ab.
15. EmOC.ti,ab.
16. emergency obstetric care.ti,ab.
17. stillb*”.ti,ab.
18. or/132-148

***HIV/AIDS***

1. exp HIV/ [MeSH]
2. AIDS.ti,ab.
3. Human Immunodeficiency Virus/ [MeSH]
4. Human Immune Deficiency Virus.ti,ab.
5. acquired immunodeficiency syndrome.ti,ab.
6. acquired immune deficiency syndrome.ti,ab.
7. HIV infections/ [MeSH]
8. Acquired Immunodeficiency Syndrome/ [MeSH]
9. HIV seropositivity/ [MeSH]
10. or/150-158

***STIs***

1. chlamydia.ti,ab.
2. gonorrhoea.ti,ab.
3. syphilis.ti,ab.
4. exp sexually transmitted diseases/ [MeSH]
5. sexually transmitted infection*.ti,ab.
6. sexually transmitted disease*.ti,ab.
7. hepatitis.ti,ab.
8. chancroid.ti,ab.
9. trichomoniasis.ti,ab.
10. human papillomavirus.ti,ab.
11. HPV.ti,ab.
12. genital wart*.ti,ab.
13. herpes.ti,ab.
14. bacterial vaginosis.ti,ab.
15. pelvic inflammatory disease/ [MeSH]
16. pelvic inflammatory disease.ti,ab.
17. PID.ti,ab.
18. mucopurulent cervicitis.ti,ab.
19. molluscum contagiosum.ti,ab.
20. lymphogranuloma venereum.ti,ab.
21. or/160-179

***Pregnancy, Maternal and Newborn Health***

1. pregnan*.ti,ab.
2. Pregnancy, Unplanned/ [MeSH]
3. Pregnancy, Unwanted/ [MeSH]
4. Pregnancy in Adolescence/ [MeSH]
5. Pregnancy outcome/ [MeSH]
6. Pregnancy complications/ [MeSH]
7. pregnancy complication*.ti,ab.
8. Maternal health/ [MeSH]
9. Maternal health.ti,ab.
10. Maternal welfare/ [MeSH]
11. Maternal welfare.ti,ab.
12. Safe motherhood.ti,ab.
13. perinatal.ti,ab.
14. Perinatal care/ [MeSH]
15. ((perinatal or antenatal or prenatal or postnatal) adj2 health).ti,ab.
16. Prenatal care/ [MeSH]
17. ((perinatal or prenatal or antenatal or postnatal) adj2 care).ti,ab.
18. Postnatal care/ [MeSH]
19. (antenat* or ante-nat* or prenat*).ti,ab.
20. (postnat* or post-nat*).ti,ab.
21. (postpart* or post-part*).ti,ab.
22. Parturition/ [MeSH]
23. Postpartum period/ [MeSH]
24. puerperium.ti,ab.
25. childbirth.ti,ab.
26. obstetrics.ti,ab.
27. Obstetrics/ [MeSH]
28. Gynecology/ [MeSH]
29. Obstetric Surgical Procedures/ [MeSH]
30. Delivery, Obstetric/ [MeSH]
31. skilled birth attend*.ti,ab.
32. EmONC.ti,ab.
33. Stillbirth/ [MeSH]
34. or/181-213

***Vaginal Injury & Fistulas***

1. fistula/ [MeSH]
2. fistula.ti,ab.
3. ((rectovaginal or urethra* or urinary tract) adj2 fistula).ti,ab.
4. exp vaginal fistula/ [MeSH]
5. (genital adj3 (trauma or injury)).ti,ab.
6. (vaginal adj3 (trauma or injury)).ti,ab.
7. or/215-220

***Gender-based violence***

1. ((gender-based or partner or family or domestic) adj2 violence).ti,ab.
2. Gender-based violence/ [MeSH]
3. violence against women.ti,ab.
4. Sex offenses/ [MeSH]
5. (sexual adj2 (abuse or violence)).ti,ab.
6. sex* crime.ti,ab.
7. Domestic Violence/ [MeSH]
8. rape.ti,ab.
9. Rape/ [MeSH]
10. Physical abuse/ [MeSH]
11. physical violence.ti,ab.
12. Intimate partner violence/ [MeSH]
13. intimate partner violence.ti,ab.
14. ((partner or spous*) abuse).ti,ab.
15. Spouse abuse/ [MeSH]
16. Sexual harassment/ [MeSH]
17. Sexual harassment.ti,ab.
18. ((abused or battered) adj2 wom#n).ti,ab.
19. Battered women/ [MeSH]
20. or/222-240

***Young people and adolescents***

1. Adolescent/ [MeSH]
2. adolescen*.ti,ab.
3. juvenile.ti,ab.
4. Minors/ [MeSH]
5. minor*.ti,ab.
6. youth.ti,ab.
7. (young adj 2 (adult or m#n or wom#n)).ti,ab.
8. (school adj6 student*).ti,ab.
9. teen*.ti,ab.
10. schoolgirl*.ti,ab.
11. schoolboy*.ti,ab.
12. adolescent sexual health.ti,ab.
13. adolescent reproductive health.ti,ab.
14. adolescent health.ti,ab.
15. youth friendly service*.ti,ab.
16. adolescent friendly service*.ti,ab.
17. adolescent health service*.ti,ab.
18. youth program*.ti,ab.
19. or/242-259

***Health services***

1. exp Reproductive health services/ [MeSH]
2. (family planning adj4 (service* or program*)).ti,ab.
3. ((sexual or reproductive) adj3 intervention).ti,ab.
4. ((sexual or reproductive) adj3 service).ti,ab.
5. or/261-264
6. 61 or 104 or 121 or 131 or 149 or 159 or 180 or 214 or 221 or 241
7. 265 or 266
8. 29 and 41 and 267
9. 260 and 268
10. Limit 269 to yr=1980-2018
